# Investigating Post-COVID-19 Risk Perception and Preventive Behaviour Among Individuals in Riyadh, Saudi Arabia

**DOI:** 10.7759/cureus.86666

**Published:** 2025-06-24

**Authors:** Roba Aljaloud, Sara Alhudaib, Shahad Alotaibi, Sara A Alsuhaibani

**Affiliations:** 1 Department of Health Science, College of Health and Rehabilitation Sciences, Princess Norah bint Abdulrahman University, Riyadh, SAU

**Keywords:** behavioural intention, covid-19, protective behaviours, risk perception, social emotion

## Abstract

Background

Behavioural intention post-COVID-19 can impact an individual’s risk perception and preventive/protective behaviour. Studies have shown that risk perception has a relationship with behavioural intentions in a way that can affect an individual's life. The aim of the study was to assess behavioural intention and preventive/protective behaviour post-COVID-19 pandemic among Saudis.

Methods

We conducted a cross-sectional (quantitative) study in Riyadh, Saudi Arabia, to forecast the perception of post-COVID-19 risk and the preventive and protective behaviours. The duration of the study was three months, starting from January to March 2023, and the sample size was 386 individuals. The target population in this study was Saudis residing in Riyadh in the age range of 18-53 years and above. The data was collected using a self-administered online questionnaire and analysed using statistical software called JMP.

Results

The total number of the study sample was 386 participants; most of the participants were women (n=316, 81.87%). The majority of participants reported taking three doses of the COVID-19 vaccine (n=288, 74.61%), and nearly 60% (n=232, 60.10%) of them reported not getting infected with COVID-19 after vaccinations. The overall regression was statistically significant (R^2^=0.478, F=175.33, p<0.0001) and it was found that social emotion and risk perception predicted behavioural intention and preventive/protective behaviour significantly. There was a moderate positive correlation between behavioural intention and the social emotion of participants (0.686). Also, there was a positive correlation relation between preventive/protective behaviour and social emotion (0.578).

Conclusion

This study revealed that social emotions and the preventive/protective intention to perform preventive/protective behaviour predict the tendency to practise preventive/protective behaviour. Risk perception also affected protective behaviour and intention.

## Introduction

Since the first outbreak of COVID-19 in late December 2019, it has developed into a significant public health issue and global concern. On March 11, 2020, the World Health Organization (WHO) proclaimed the virus outbreak a worldwide pandemic [1]. The preventive/protective behaviours that societies must adopt during the COVID-19 pandemic to limit its spread are washing hands frequently, using hand sanitizers, avoiding crowded places, covering the mouth and nose when coughing, and not touching the face when the hand is unclean [2]. The pandemic got under control considerably more rapidly thanks to the rise in the number of people following precautions in countries like China, Korea, and Japan [3]. The incidence of COVID-19 pandemic-related cases is still increasing exponentially in countries where such measures are not mandatory [4]. Vaccinations against COVID-19 were also important in controlling its spread around the world [5]. It has also been proven that receiving the COVID-19 vaccine reduces the number of infections and, thus, the number of deaths [6]. To reduce an individual’s chance of infection in the post-COVID-19 period, individuals needed to incorporate risk prevention/protection behaviour into their everyday routines and work practices [1]. A previous study about COVID-19 showed that higher perceptions of the risk of contracting COVID-19 infection and higher perceptions of the severity of COVID-19 were linked to a higher likelihood of engaging in preventative/protective behaviours [7]. A study conducted in Ireland investigated COVID-19 risk perception and preventive/protective behaviours among university students. They demonstrated in their findings that students' risk perceptions were high, and they exhibited high adherence to preventive and protective behaviours, which encouraged them to take the COVID-19 vaccine [8]. After the outbreak of COVID-19 pandemic, people have been significantly impacted when it comes to risk perception [9]. A study was conducted in Japan to investigate factors associated with the risk perception of COVID-19 infection and severe illness [10]. They found that those who regarded the COVID-19 vaccination risk as being higher were more likely to also consider the risk of infection and serious illness as being higher [10]. It's interesting to note that those who perceived a higher risk of infection were more likely to say they got their information from medical professionals, whereas those who perceived a lower risk were more likely to say they got it from the government or the Internet; similarly, those who perceived a lower risk of being seriously ill were more likely to say they got it from the Internet. The likelihood of both risk perception of infection and likelihood of developing serious illness decreases with increasing government trust as a source of COVID-19 information [10]. We aim to explore the effects of social emotion, risk perception of behaviour intention, and prevention/protection behaviours post- COVID-19 in our study among Saudis in Riyadh.

Theoretical framework

We used the theoretical framework based on the protective action decision model (PADM) and the risk perception emotional component where we followed a study conducted by Shi et al. [1]. According to previous research, PADM is a multistage model based on results from studies about responses to environmental hazards and potential harm [11]. Hence, PADM combines the gathering of information produced from social and environmental indicators with messages that social sources communicate to persons who are at risk through communication channels [12]. Moreover, PADM defines three key pre-decision processes that come before all other processing, including reception, attention, and comprehension of warnings or exposure, attention, and interpretation of environmental and social indicators. The revised model identifies two core perceptions, threat perceptions, and protective action perceptions. A behavioural response is the result of the protective action decision-making process, situational facilitators, and barriers [13].

Risk Perception

It is founded on the perception of the risks in the immediate surroundings and reflects the individual's subjective or direct assessment of potential risks, such as an adverse event [12]. Risk perception is divided into individual and social aspects. The individual aspect is used to assess the potential risk of infection for the individual, and the social aspect is used to assess the potential risk of infection for other individuals or the community [1].

Social Emotion

Social emotion is the social signaling of all of one's emotions to others and the externalization of one's own emotions [14]. The risk perception emotion model defines social emotion as the individual's subjective evaluation and psychological sentiments surrounding their current social environment [14]. According to the model, an individual's perceived susceptibility based on emotion is a variable that causes a person to produce behavioural responses in the face of threats or harm, and the corresponding affective association changes the behavioural response effectively. Different emotional states may impact behavior in various ways, according to some experts [1].

Protective Behaviour

An individual's willingness to engage in adaptive behaviour is subjectively evaluated by the decision they make to engage in protective behaviour (protective behavioural intention). According to PADM, through an individual's behavioral intentions, their perception of risk may influence their protective behaviour directly or indirectly. It is also possible to predict the adaptive behaviour of individuals by perceiving the risks and their behavioural intentions toward them. This relationship has also been confirmed in many studies on health-related behaviours [1].

Objectives

1. Assess the level of intention to perform preventive/protective behaviour among individuals in Riyadh.

2. Assess practicing preventive/protective behavior post-COVID-19 among individuals in Riyadh.

## Materials and methods

Study design, study area, study population and sampling

A cross-sectional (quantitative) study was applied to predict the post-COVID-19 risk perception and preventive/protective behaviours in Riyadh, Saudi Arabia. An online questionnaire was sent to participants via social network channels, including WhatsApp and Twitter, and the goal was to investigate post-COVID-19 risk perception and protective behaviours. This investigation was carried out from January to March 2023. The inclusion criteria included Saudi residents in Riyadh, both men and women, who ranged in age from 18 to 53 years and above. Saudis do not live in Riyadh, and the age group under 18 was excluded from the study.

Sample technique and sample size

Our study used a convenient sampling technique. We calculated the sample size to be 385 participants using the open-source OpenEpi software, version 3.01 (OpenEpi, 2022). The largest possible sample size can be determined via a cross-sectional study, considering a design effect of 1.5 and a prevalence of 50%. We sent a link to many individuals, but closed the survey once we reached the required sample size.

Data collection and list of variables

The research team created a questionnaire from a previous study by Shi et al. (2022) and translated it into an Arabic questionnaire. The team then uploaded the survey to an online Google form to gather data for this study (Appendix). The questionnaire contains three parts. The first part consists of six questions about sociodemographic characteristics, including gender, group age, education level, marital status and occupation. The second part consists of five questions about infection with COVID-19 and vaccine-related characteristics. The third part consists of three subscales: pandemic risk perception, social emotion, and preventive/protective behaviours. All questions were closed-ended questions with multiple response types, such as 'yes' or 'no', and Likert scale questions. We also consulted an expert for the questionnaire's validation. The reliability of questions was tested using Cronbach’s alpha to examine the internal consistency of questions. Cronbach’s alpha test results from our questionnaire were α=0.5921 for risk perception and 0.9085 for preventive/protective behaviours.

Sociodemographic Characteristics

Sociodemographic characteristics identified eight questions, which include nationality, region in Saudi Arabia, gender, age, level of education, marital status, occupation, and household monthly income level. The questions were multiple choice and close-ended.

The Protective Action Decision Model and Risk Perception Model

The three sections guided by the PADM and risk perception model assessed four items: (1) social emotion (independent variables); (2) behaviour intention (dependent variable); (3) risk perception (independent variable); and (4) protective behaviour (dependent variables). Likert scales (strongly agree/agree/ neutral/disagree/strongly disagree) were used for assessing responses to the close-ended questions.

Social Emotion

It is the social signalling of all one’s emotions to others and the externalisation of one’s own emotions. Four items measured social emotions: (1) Is it important to be protected from COVID-19 at all times? (yes/no/I don’t know); (2) Reduced going to crowded places (strongly agree/ agree/neutral/agree/strongly disagree); (3) I reduced going to family meetings for fear of infection (strongly agree/agree/neutral/disagree/strongly disagree); (4) If I have to attend a family meeting or because of work, I take the necessary measures and precautions for that (strongly agree/agree/neutral/disagree/strongly disagree).

Behaviour Intention

Four items were used to measure behaviour intention toward COVID-19: (1) I intend to take any additional doses of COVID-19 vaccinations in the future (strongly agree/agree/neutral/disagree/strongly disagree); (2) I intend to continue wearing face masks in public for the next three months (strongly agree/agree/neutral/ disagree/strongly disagree); (3) I intend to continue to go to public places less often for the next three months (strongly agree/agree/neutral/disagree/strongly disagree); (4) I intend to continue going to family and friend meetings less often for the next three months (strongly agree/agree/neutral/disagree/strongly disagree).

Risk Perception

It reflects the individual’s subjective or direct assessment of potential risk, which was assessed by six items: (1) Timely COVID-19 information made me care (strongly agree/agree/neutral/disagree/strongly disagree); (2) I think I might have COVID-19 (strongly agree/agree/neutral/disagree/strongly disagree); (3) I think I might be at a higher risk of catching COVID-19 (strongly agree/agree/neutral/disagree/strongly disagree); (4) I think someone around me might have COVID-19 (strongly agree/agree/neutral/disagree/strongly disagree); (5) The COVID-19 virus is still circulating (strongly agree/agree/neutral/disagree/strongly disagree); (6) COVID-19 poses danger to the society (strongly agree/agree/neutral/disagree/strongly disagree).

Protective Behaviour

An individual’s willingness to engage in adaptive behaviour. Protective behaviour was assessed by eight items: (1) I am still taking precautions for COVID-19 (strongly agree/agree/neutral/disagree/strongly disagree); (2) I will implement the preventive/protective measures for COVID-19 during the next three months (strongly agree/ agree/neutral/disagree/strongly disagree); (3) Take more effective measures to prevent/protect against COVID-19 (strongly agree/agree/neutral/disagree/strongly disagree); (4) Wash my hands regularly and keep hands clean (strongly agree/agree/neutral/disagree/strongly disagree); (5) I cover my mouth when I cough or sneeze (strongly agree/agree/neutral/disagree/strongly disagree); (6) Wear face masks in crowded places (strongly agree/agree/neutral/disagree/strongly disagree); (7) I clean/disinfect frequently touched surfaces such as doorknobs (strongly agree/agree/neutral/disagree/strongly disagree); (8) Ensure social distancing in public places (strongly agree/agree/neutral/disagree/strongly disagree).

Vaccinations and Infection with COVID-19

Vaccinations and infection with COVID-19 were assessed with five items: (1) How many doses of COVID-19 vaccinations did you get? (I didn't get any doses, one dose, two doses, three doses, or four doses; close-ended questions); (2) Have you been infected with the coronavirus after taking vaccinations? (No, I have not been infected with the coronavirus; yes, I was hit once; yes, I was infected more than once; close-ended questions); (3) Have you been infected with the coronavirus during the past two months? (Yes/No; close-ended questions); (4) I intend to take any additional doses of COVID-19 vaccinations in the future (strongly agree/ agree/neutral/disagree/strongly disagree); (5) Is it crucial to maintain constant protection against COVID-19? (Yes/ No/I don’t know; close-ended questions).

Participants in the study and data analysis plan

The participants were volunteers, unpaid, and anonymous from the main Riyadh regions. Using an online questionnaire, we targeted Saudi residents. To analyse data, the statistical package JMP (SAS Institute, Cary, NC, USA) was used, with descriptive and analytical statistics. We conducted a linear regression coefficient test and correlation to assess the relationship between risk perception, preventive and protective behaviours, and sociodemographic factors. In our study, the 0.05 level was used as a cutoff point of significance. Linear regression analysis was used to predict if social emotions and high-risk perception predict preventive/protective behaviour. This procedure estimates the strength of the association between each independent variable and the dependent variable.

Ethical consideration 

The College of Health and Rehabilitation Sciences at Princess Norah bint Abdulrahman University (22-1040) granted ethical approval. All participants were informed about the study's objectives and it was also clarified that their participation is voluntary.

## Results

Table 1 demonstrates the sociodemographic characteristics of the sample studied. The total number of the study sample was 386 participants (N=386). Of them, 70 were men and 316 were women. In the study, 165 (43.26%) participants were aged between 18 and 24 years while only 37 (9.59%) were aged 53 years and older. Looking at marital status, we found that 180 (46.63%) of the participants were married, whereas nine (2.33%) were divorced or widowed. Moreover, half of the participants in this study had a bachelor’s degree (n=195, 50.52%), whereas only eight (2.07%) had secondary or primary education. When looking at employment status, most of the participants (n=163, 42.23%) were students, and 94 (24.35%) were employees in both sectors, and 71 (18.39%) were unemployed.

**Table 1:** Sociodemographic descriptive statistics of participants (N=386)

Variable	Category	Frequency	Percent
Age (years)	18-24	165	43.26
	25-31	49	12.69
	32-38	44	12.40
	39-45	46	11.92
	46-52	43	11.14
	53+	37	9.59
Gender	Male	70	18.13
	Female	316	81.87
Education level	Intermediate or less	8	2.07
	Secondary - Diploma	143	37.05
	Bachelor's	195	50.52
	Postgraduate studies	40	10.36
Employment status	Student	163	42.23
	Employee (public sector)	62	16.06
	Employed (private sector)	32	8.29
	Unemployed	71	18.39
	Retired	28	7.77

Table 2 demonstrates the intention toward COVID-19 vaccinations. Seven (1.81%) participants did not get any dose, while 288 (74.61%) got three doses. The majority of the participants did not get infected with COVID-19 after vaccination while 133 (34.46%) were infected with COVID-19 after vaccination. Moreover, 127 (32.90%) participants reported neutral towards additional doses of COVID-19 vaccination.

**Table 2:** Participant's intention towards various doses of COVID-19 vaccination

Characteristics	Category	Frequency	Total percentage (%)
How many doses of COVID-19 vaccinations did you get?	I didn't get any dose	7	1.81
	One dose	10	2.59
	Two doses	78	20.21
	Three doses	288	74.61
	Four doses	3	0.78
Have you been infected with coronavirus after taking vaccinations?	No, I did not have the coronavirus	232	60.10
	Yes, I was hit once	133	34.46
	Yes, I was injured more than once	21	5.44
I intend to take any additional doses of COVID-19 vaccinations in the future	Strongly agree	12	3.11%
	Agree	48	12.44
	Neutral	127	32.90
	Disagree	88	22.80
	Strongly disagree	111	28.76

**Figure 1:**
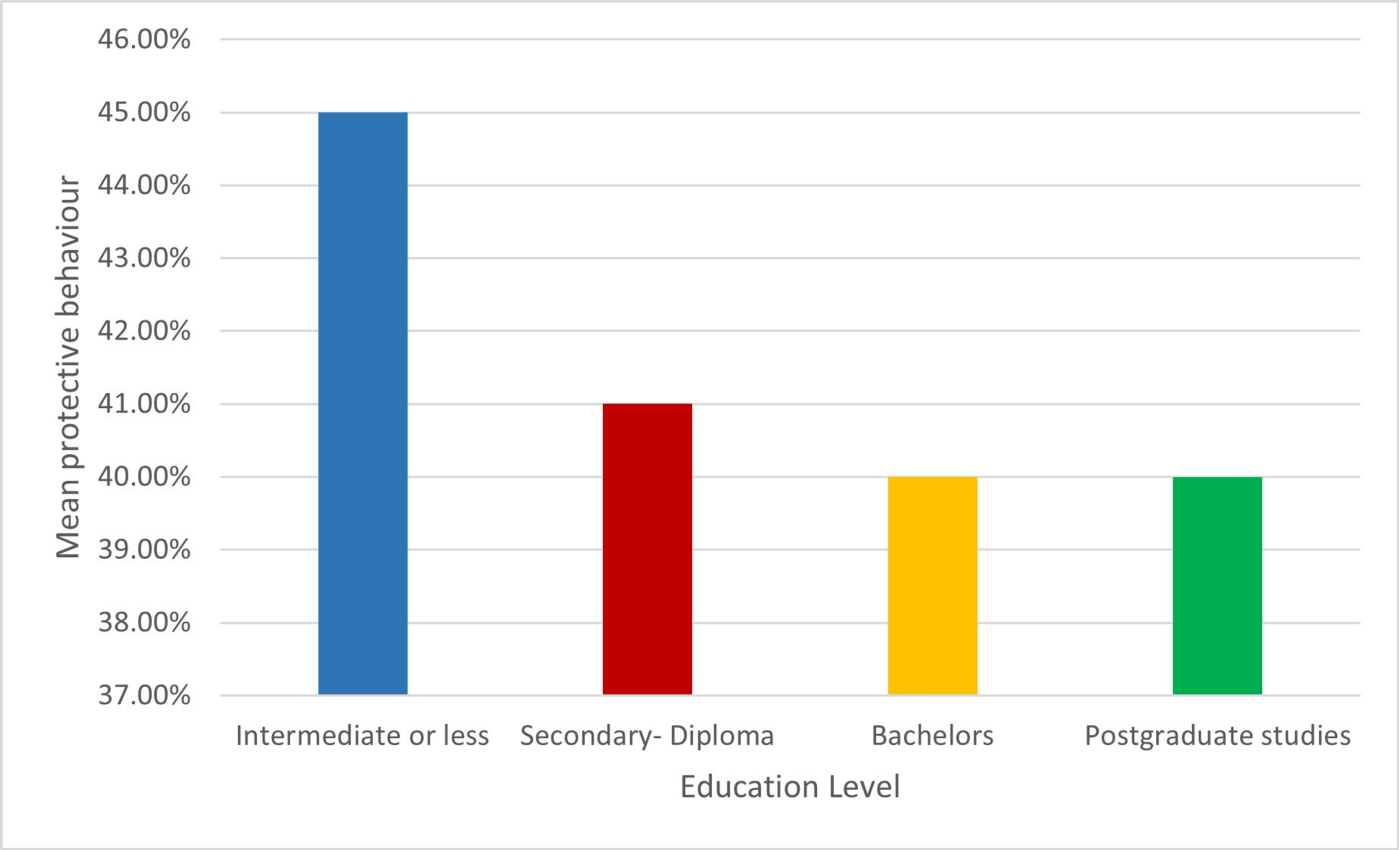
Mean of protective/protective behavior and educational level (n=386) Image credit: Ms. Roba Aljaloud

Figure 1 shows the mean of protective/protective behaviour and education level; the participants who had intermediate or less education committed more to post-COVID-19 protective/protective behaviour.

**Figure 2:**
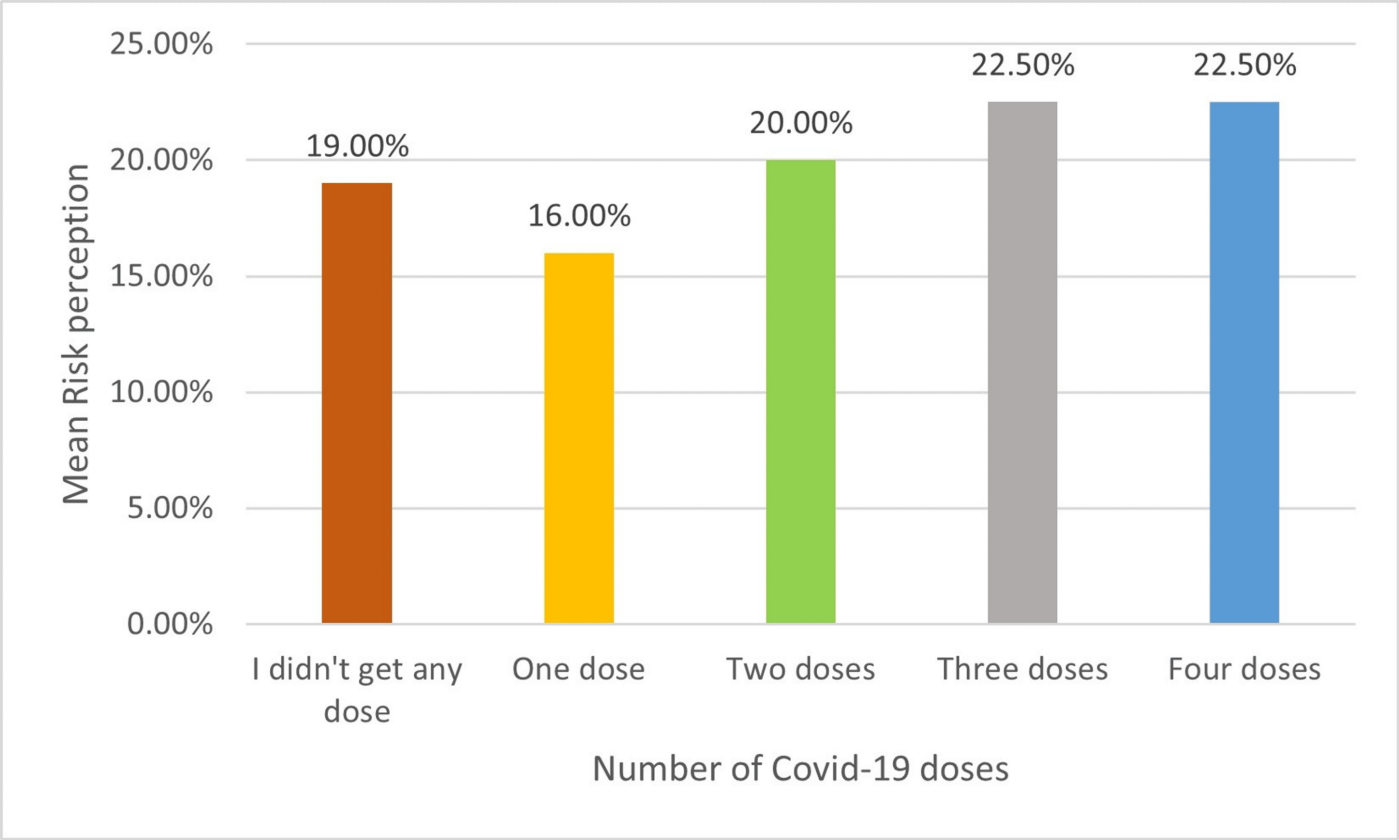
Mean of risk perception and number of COVID-19 doses (n=386) Image credit: Ms. Roba Aljaloud

Figure 2 demonstrates the mean of risk perception and the number of doses of COVID-19 vaccination, showing that the single dose has low risk perception, while high risk perception is in those who had four doses.

A linear regression analysis was performed to predict the behavioural intention to post-COVID-19 preventive/protective behaviour among the study population in Riyadh (Table 3). Social emotion and risk perception were entered as independent variables. Both variables were positively correlated with intention and were significant (F=175.33, p<0.0001); social emotion and risk perception accounted for about 48% of the variance in intention. Social emotion had regression weight on intention (β=0.79, p<.0001) and risk perception (β=0.77, p=0.023) were also studied. We found a moderate positive correlation relationship between the behavioural intention and the social emotion of participants (0.6863).

**Table 3:** Multiple linear regression analysis of intention to post-COVID-19 behavior Note: R^2^= 0.477

Term	Estimate	Std Error	t Ratio	Prop>t	r
Social emotion	1.1475	0.090	12.69	< .0001>	0.5781
Risk perception	0.1955	0.067	2.90	0.0039*	0.2732

Table 4 shows the results of linear regression analysis used to predict post-COVID-19 preventive/protective behaviour among the study population in Riyadh. Preventive/protective behaviour was entered as the dependent variable and variables that had a significant relationship with preventive/protective behaviour (social emotion, risk perception) were entered as independent variables (F= 102.45, p<.0001). Social emotion and risk perception accounted for about 35% of the variance in protective behaviour. Social emotion had the strongest regression weight on preventive/protective behaviour (β=0.20, p<.0001), followed by risk perception (β=0.15, p=0.003). Also, we found a positive correlation relationship between preventive/protective behaviour and social emotion (0.578).

**Table 4:** Multiple linear regression analysis of preventive/protective behavior Note: R^2^= 0.348548

Variables	Estimate	Std Error	t Ratio	Prop>t	r
Social emotion	0.7868	0.0455	17.26	<.0001	0.6863
Risk perception	0.0769	0.0339	2.27	0.0239*	0.2681

## Discussion

The purpose of this study was to explore the relationship between risk perception and intention of preventive/protective behaviours based on the PADM theory and adding a risk perception component. The current study, conducted on 386 Saudi participants who live in Riyadh and are aged 18 to over 53 years old, aimed to understand which variables influenced the intention towards preventive/protective behaviour and practising preventive/protective behaviour post-COVID-19.

Firstly, our investigation showed that individuals with low levels of education had a higher level of practising preventive/protective behaviours, and we did not find differences between Saudi women and men in compliance with preventive/protective behaviours. Similarly, other studies showed that those with higher levels of education practiced more preventive hygiene; those with less education did the opposite [15, 16]. This outcome makes sense since education raises health awareness and people become more likely to be health literate. The same holds true for the lower level of schooling. They might not give health education and public health top priority since they might not value them. A study conducted in Germany revealed that both highly educated men and women had higher preventive/protective behaviours, unlike the published results regarding differences in risk perception, knowledge, and behaviour by education level. Also, the study showed the compliance differences towards preventive/protective behaviours: women were found to have higher handwashing compliance, while compliance with maintaining distance was found to be higher in men [17].

Secondly, our study results demonstrated that participants who reported taking COVID-19 vaccines had a higher risk perception. Our results were aligned with a study conducted in Ireland by Borges and Byrne, which revealed that showing a higher level of risk perception increased preventive/protective behaviours such as taking the vaccine [8]. The majority of participants took three doses; the response may be influenced by the Ministry of Health registration starting in February 2022, receiving the booster dose requirement for getting an immune status on the Tawakkalna application, which is a prerequisite to entering any place, activity or travelling [18].

The regression analysis revealed that both variables significantly predicted behavioural intentions, accounting for nearly 48% of the variance. These findings suggest that a considerable portion of individuals’ intentions to engage in preventive behaviours can be explained by how emotionally connected they feel to the social consequences of COVID-19, as well as how they perceive the associated risks. Interestingly, the strongest predictor turned out to be social emotion. This emphasises the power of emotional engagement, such as empathy, fear of harming others, or community solidarity, in motivating protective behaviours [19]. A moderate positive correlation between social emotion and behavioural intention further supports this finding, suggesting that individuals who experience stronger emotional responses to the social impact of COVID-19 are more likely to intend to adopt protective actions like mask-wearing, hand hygiene, and physical distancing [20].

These findings align with previous research showing that emotional factors often play a stronger role than purely cognitive assessments in health-related decision-making. For instance, studies on the Health Belief Model and Protection Motivation Theory suggest that both perceived risk and emotional cues (such as fear or concern for others) are crucial in shaping behaviour change during pandemics [19]. Particularly during global health crises, social emotions such as moral responsibility, compassion, and solidarity become salient motivators for collective action [21].

The significance of risk perception is consistent with findings from various behavioural health studies that identify perceived susceptibility and severity as important precursors to adopting preventive measures [22,23]. However, the slightly lower beta coefficient and p-value for risk perception in this study may indicate that people's actions are influenced not just by their understanding of the risks but are more strongly motivated by their feelings and social connections during the pandemic experience [22,24,25].

These insights have important public health implications. While traditional campaigns often focus on raising awareness of the risks of infection, our results suggest that integrating emotional narratives and social appeal - such as emphasising family protection, contributing to community safety, or avoiding regret - could significantly boost behavioural compliance [26,27]. Campaigns that humanise the consequences of inaction and evoke emotional engagement may be more effective for sustaining long-term protective behaviours [28].

Furthermore, the findings may reflect cultural factors specific to Riyadh and the broader Saudi context, where collectivist values and a strong sense of social responsibility may amplify the effects of social emotions on health behaviours [29]. Future studies could explore how cultural norms moderate these relationships and whether similar patterns emerge in individualistic societies.

There are a few limitations of this study. The research was confined only to Saudis who live in the city of Riyadh. The majority of the participants were women, and the percentage of men was very low. The study's reliance on convenient sampling and online-only distribution introduces bias by potentially excluding older or less tech-savvy individuals. This limitation may affect the generalisability of the findings. Additionally, incorporating face-to-face interviews or phone surveys could help reach individuals who may not have access to or be comfortable with online surveys. Such an approach would enhance the inclusivity and diversity of the study's sample. It is important to note that the results may not be representative of the entire Saudi population due to the limited sample size and demographic restrictions. Future studies should aim to include a more diverse sample from various regions in Saudi Arabia for a more comprehensive understanding of the topic.

## Conclusions

The findings of our study showed that social emotions and risk perception lead participants to preventive/protective behavioral intention and to perform preventive/protective behavior, and social emotion and risk perception predict the intention to practice preventive/protective behavior among target populations. Social emotion has the strongest weight on behavioral intention and protective behavior. Therefore, there is a need to conduct programs and campaigns targeted at social emotion factors because it has the strongest influence on protective behaviours. Lastly, there is a need for more studies to investigate post-COVID-19 risk perception and preventive/protective behaviour to include all regions of Saudi Arabia.

## Appendices

Questionnaire 

**Table 5:** Section on sociodemographic questions

Variable	Options
Nationality	Saudi
	Non-Saudi
Do you live in Riyadh?	Yes
	No
Gender	Male
	Female
Age	18-24 years old
	25-31 years old
	32-38 years old
	39-45 years old
	46-52 years old
	53-59 years old
	60 years and above
Education level	Does not have an elementary school certificate
	Elementary - Intermediate
	Secondary - Diploma
	Bachelor's degree
	Postgraduate studies
Marital status	Single
	Married
	Separate
	A widower
What is your profession?	Student
	Government sector employee
	Private sector employee
	Entrepreneurship
	Retired
	Unemployed
	Other
Family income level (Saudi Riyal (SAR))	Less than 4000
	From 4000-7000
	From 7001-10000
	From 10001-13000
	From 13001-16000
	From 16001-19000
	From 19001-22000
	More than 22000

In the following section, we ask a set of questions about your infection with the COVID-19 virus and the vaccinations that you got.

**Table 6:** Section on vaccinations and COVID-19 infection

Variable	Options
How many doses of COVID-19 vaccinations did you get?	I did not get any dose
	Single dose
	Two doses
	Three doses
	Four doses
Have you been infected with coronavirus after taking vaccinations?	No, I have not been infected with the Corona virus
	Yes, I was hit once
	Yes, I was infected more than once
have you been infected with corona during the last two months?	Yes
	No
I intend to take any additional doses of COVID-19 vaccinations in the future	Strongly agree
	Agree
	Neutral
	Disagree
	Strongly disagree
Is it important to be protected from COVID-19 at all times?	Yes
	No
	I don't know

In the following section, we ask a set of questions that focus on risk perception about COVID-19*.*

**Table 7:** Risk perception section

Risk perception	Strongly agree	Agree	Neutral	Disagree	Strongly disagree
Timely COVID-19 information made me care					
I think I might have COVID-19					
I think I might be at a higher risk of catching COVID-19					
I think someone around me might have COVID-19					
The COVID-19 virus is still circulating					
COVID-19 poses a danger to society					

In the next section, we ask a set of questions about your current and future preventive behaviours.

**Table 8:** Preventive behaviours section

Preventive Behaviors	Strongly Agree	Agree	Neutral	Disagree	Strongly Disagree	
I am still taking precautions for COVID-19						
I will implement the preventive measures COVID-19 during the next three months						
Take more effective measures to prevent COVID-19						
Wash my hands regularly and keep hands clean						
I cover my mouth when I cough or sneeze						
Wear face masks in crowded places						
I clean/disinfect frequently touched surfaces such as doorknobs						
Reduced going to crowded places						
I reduced going to family meetings for fear of infection						
If I have to attend a family meeting or because of work, I take the necessary measures and precautions for that						
Ensure social distancing in public places						
I intend to continue wearing face masks in public places for the next three months						
I intend to continue to go to public places less often for the next three months						
I intend to continue going to family and friend meetings less often for the next three months						

## References

[1] Shi G, Zhong X, He W, Liu H, Liu X, Ma M (2021). Factors influencing protective behavior in the post-COVID-19 period in China: a cross-sectional study. Environ Health Prev Med.

[2] Aschwanden D, Strickhouser JE, Sesker AA, Lee JH, Luchetti M, Terracciano A, Sutin AR (2021). Preventive behaviors during the COVID-19 pandemic: associations with perceived behavioral control, attitudes, and subjective norm. Front Public Health.

[3] Holbig H (2022). Navigating the dual dilemma between lives, rights and livelihoods: COVID-19 responses in China, Singapore, and South Korea. Z Vgl Polit.

[4] Güner R, Hasanoğlu I, Aktaş F (2020). COVID-19: prevention and control measures in community. Turk J Med Sci.

[5] Baba IA, Humphries UW, Rihan FA (2023). Role of vaccines in controlling the spread of COVID-19: a fractional-order model. Vaccines (Basel).

[6] Coccia M (2022). Optimal levels of vaccination to reduce COVID-19 infected individuals and deaths: a global analysis. Environ Res.

[7] Hilverda F, Vollmann M (2021). The role of risk perception in students' COVID-19 vaccine uptake: a longitudinal study. Vaccines (Basel).

[8] Borges J, Byrne M (2022). Investigating COVID-19 risk perception and preventive behaviours in third-level students in Ireland. Acta Psychol (Amst).

[9] Arefi MF, Babaei AP, Barzanouni S, Ebrahimi S, Salehi AR, Khajehnasiri F, Poursadeghian M (2022). Risk perception in the COVID-19 pandemic; a health promotion approach. J Educ Health Promot.

[10] Adachi M, Murakami M, Yoneoka D (2022). Factors associated with the risk perception of COVID-19 infection and severe illness: a cross-sectional study in Japan. SSM Popul Health.

[11] Stander B, Zhang H, Lin C-C, Wu H-C, Murphy H, Huang S-K (2025). Risk perception and the U-pattern of protective action changes: analyzing responses to rare tornado threats. Int J Disaster Risk Reduct.

[12] Guo Y, An S, Comes T (2022). From warning messages to preparedness behavior: the role of risk perception and information interaction in the Covid-19 pandemic. Int J Disaster Risk Reduct.

[13] Lindell MK, Perry RW (2012). The protective action decision model: theoretical modifications and additional evidence. Risk Anal.

[14] Hopfer S, Fields EJ, Lu Y (2021). The social amplification and attenuation of COVID-19 risk perception shaping mask wearing behavior: a longitudinal twitter analysis. PLoS One.

[15] Alves RF, Samorinha C, Precioso J (2020). Knowledge, attitudes and preventive behaviors toward COVID-19: a study among higher education students in Portugal. J Health Res.

[16] Apanga PA, Kamal Lettor IB, Akunvane R (2020). Practice of COVID-19 preventive measures and its associated factors among students in Ghana. Am J Trop Med Hyg.

[17] Rattay P, Michalski N, Domanska OM, Kaltwasser A, De Bock F, Wieler LH, Jordan S (2021). Differences in risk perception, knowledge and protective behaviour regarding COVID-19 by education level among women and men in Germany. Results from the COVID-19 Snapshot Monitoring (COSMO) study. PLoS One.

[18] (2023). Kingdom of Saudi Arabia, Ministry of Interior: Taking a booster dose is a requirement for individuals who have spent 8 months since obtaining the second dose to obtain an “immune” status in Twakkalna. https://www.moi.gov.sa/wps/portal/Home/Home/dp-home/!ut/p/z0/fYyxDoIwGIRfBQZG8v9UKDpWB1EiiSEx0KVpsIEaaAMS8PEF3F0ud99dDjiUMY3CmAQUCuBGTrqWo7ZGtksuORWH-_mUJCFJMdsjskvGHukNd5hTyJWBK_D_o-VFv_qeM-CVNaP6jFB0VjtPPUvj4Wob2ylnK83oyMHDFXho1Pz-6QKrRk9qm4uVCIz8gPgESaCFHKDjx7ZmrvsF2MWw2A!!/..

[19] Marikyan D, Papagiannidis S (2023). Protection motivation theory: a review. TheoryHub Book: This handbook is based on the online theory resource: TheoryHub.

[20] Eddy CM (2021). The social impact of COVID-19 as perceived by the employees of a UK mental health service. Int J Ment Health Nurs.

[21] Sels L, Tran A, Greenaway KH, Verhofstadt L, Kalokerinos EK (2021). The social functions of positive emotions. Current Opinion in Behavioral Sciences.

[22] Savadori L, Lauriola M (2020). Risk perception and protective behaviors during the rise of the COVID-19 outbreak in Italy. Front Psychol.

[23] Šuriņa S, Martinsone K, Perepjolkina V (2021). Factors related to COVID-19 preventive behaviors: a structural equation model. Front Psychol.

[24] Qin H, Sanders C, Prasetyo Y, Syukron M, Prentice E (2021). Exploring the dynamic relationships between risk perception and behavior in response to the Coronavirus Disease 2019 (COVID-19) outbreak. Soc Sci Med.

[25] Hamiduzzaman M, Siddiquee N, McLaren H, Tareque MI, Smith A (2022). Risk perception and health precautions towards COVID-19 among older culturally and linguistically diverse adults in South Australia: a cross-sectional survey. J Multidiscip Healthc.

[26] Avelino-Silva VI, Ferreira-Silva SN, Soares ME (2023). Say it right: measuring the impact of different communication strategies on the decision to get vaccinated. BMC Public Health.

[27] Liu J, Yang X, Lu Y, Zheng X (2022). The joint effects of social norm appeals and fear appeals in COVID-19 vaccine campaign posters on self-perceived communication quality and vaccination intention. Front Psychol.

[28] Williamson KA, Thulin E (2022). Leveraging emotion-behavior pathways to support environmental behavior change. Ecology and Society.

[29] Al-Ghuraibi MA, and Aldossry TM (2022). Social Stigma as an outcome of the cultural repercussions toward COVID-19 in Saudi Arabia. Cogent Social Sciences.

